# Targeting *Catenibacterium mitsuokai* with icariin modulates gut microbiota and improves hepatic lipid metabolism in intrauterine growth restriction

**DOI:** 10.1093/ismejo/wraf141

**Published:** 2025-07-03

**Authors:** Yusen Wei, Jiangdi Mao, Wenjie Tang, Yanfei Ma, Jiachen Li, Songtao Su, Zhixiang Ni, Jinhong Wu, Daren Liu, Haifeng Wang

**Affiliations:** The Key Laboratory of Molecular Animal Nutrition, Ministry of Education, College of Animal Science, Zhejiang University, Hangzhou 310058, China; The Key Laboratory of Molecular Animal Nutrition, Ministry of Education, College of Animal Science, Zhejiang University, Hangzhou 310058, China; The Key Laboratory of Molecular Animal Nutrition, Ministry of Education, College of Animal Science, Zhejiang University, Hangzhou 310058, China; The Key Laboratory of Molecular Animal Nutrition, Ministry of Education, College of Animal Science, Zhejiang University, Hangzhou 310058, China; The Key Laboratory of Molecular Animal Nutrition, Ministry of Education, College of Animal Science, Zhejiang University, Hangzhou 310058, China; The Key Laboratory of Molecular Animal Nutrition, Ministry of Education, College of Animal Science, Zhejiang University, Hangzhou 310058, China; The Key Laboratory of Molecular Animal Nutrition, Ministry of Education, College of Animal Science, Zhejiang University, Hangzhou 310058, China; The Second Affiliated Hospital of Zhejiang University, Department of Hepatobiliary and Pancreatic Surgery, Hangzhou 310009, China; The Second Affiliated Hospital of Zhejiang University, Department of Hepatobiliary and Pancreatic Surgery, Hangzhou 310009, China; The Key Laboratory of Molecular Animal Nutrition, Ministry of Education, College of Animal Science, Zhejiang University, Hangzhou 310058, China

**Keywords:** IUGR, icariin, microbiota, lipid metabolism, Catenibacterium, piglet

## Abstract

Male offspring with intrauterine growth restriction (IUGR) exhibit more pronounced hepatic lipid metabolism abnormalities than females, necessitating earlier intervention. Icariin (ICA) has been shown to effectively modulate hepatic lipid metabolism in male piglets with IUGR. However, the role of gut microbiota in this process remains to be elucidated. This study aimed to explore the influence of gut microbiota on ICA-induced enhancement of hepatic lipid metabolism. By examining changes in microbiota composition and hepatic lipid metabolism following ICA intervention, the study demonstrated an association between microbial alterations and hepatic lipid regulation through fecal microbiota transplantation. The impact of *Catenibacterium* on gut microbiota structure and hepatic lipid metabolism was assessed *in vivo*, and the direct effect of ICA on *Catenibacterium* was explored *in vitro*. Results revealed that ICA intervention modified fecal, ileal, and colonic microbiota in male piglets with IUGR, enhanced gut morphology and barrier function, and normalized the expression of hepatic peroxisome proliferator-activated receptor signaling pathway-related genes. Fecal microbiota transplantation from piglets with IUGR impaired intestinal barrier function and led to hepatic lipid deposition, whereas transplantation from ICA-treated donors showed no pathological changes, an outcome associated with reduced abundance of *Catenibacterium*. Mechanistically, ICA inhibits adenosine triphosphate synthesis to suppress *Catenibacterium*, remodels gut microbiota, reduces lipopolysaccharide production and translocation, and activates the hepatic PPARα/CD36 axis. In conclusion, ICA intervention alleviates hepatic lipid metabolic disorders in male offspring with IUGR by suppressing *Catenibacterium*, restoring gut microbial balance, and enhancing intestinal barrier integrity to limit lipopolysaccharide translocation.

## Introduction

Intrauterine growth restriction (IUGR) is a significant global public health concern, affecting ~10%–15% of newborns [1]. Epidemiological studies indicate that IUGR offspring is more prone to develop metabolic diseases in adulthood, with males exhibiting greater susceptibility than females [1]. IUGR piglets serve as an optimal animal model for studying human medical IUGR due to their numerous physiological similarities, particularly the more severe liver injury and metabolic impairments commonly observed in males [2]. Our previous studies have shown that male IUGR piglets require more urgent early interventions than females [2], primarily due to the disrupted hepatic lipid metabolism, which induces excessive inflammation and leads to long-term impairments in liver function and growth performance. The peroxisome proliferator-activated receptor (PPAR) signaling pathway, which plays a crucial role as a link between lipid signaling and inflammatory responses, has garnered widespread attentions for its dysfunction in the liver of IUGR piglets, characterized by the reduced mRNA levels of PPARA and the elevated mRNA levels of PPARG and CD36, collectively indicating the disrupted lipid metabolism [3–5]. Icariin (ICA), a flavonol glycoside derived from *Epimedium* species, has been extensively studied for its anti-inflammatory, antioxidant, and regulatory effects on lipid metabolism, including the suppression of lipopolysaccharide (LPS)-induced inflammation [6], inhibition of foam cell formation via downregulation of CD36 [7], and prevention of hepatic steatosis through modulation of the PPAR signaling pathway [8]. In our previous work, ICA effectively activate hepatic PPARα expression in male IUGR piglets, leading to significant lipid-lowering effects [2].

In our previous study, ICA intervention in male IUGR piglets significantly reduced hepatic levels of pro-inflammatory cytokines [2]. The inflammatory response in the liver of IUGR piglets might be associated with the upregulated mRNA levels of *TLR4* [9]. As a receptor that recognizes microbial danger signals like LPS, the function of TLR4 is closely linked to changes in the gut microbiota [10]. Changes in the composition of the gut microbiota or the impaired intestinal permeability could lead to the translocation of microorganisms or harmful metabolites to the liver via the portal vein, which in turn affects liver function [11]. Recent research has shown that the gut microbiota not only plays a crucial role in maintaining normal gut function but also is closely linked to several extra-intestinal diseases, including liver diseases [12]. Consequently, modulating the gut microbiota composition holds considerable promise for mitigating liver-related diseases. Previous studies have demonstrated that the gut microbiota composition in both the small and large intestines is significantly altered in IUGR piglets compared with the normal [13, 14], alongside a disruption in barrier function [15, 16]. Furthermore, nutritional interventions are bound to induce changes in the composition or function of the gut microbiota [17], highlighting the potential role of the nutritional intervention targeting gut microbiota in improving hepatic lipid metabolism in male IUGR piglets.

Given these findings, this study focuses on male IUGR piglets to explore the effects of ICA intervention on gut microbiota composition. Through fecal microbiota transplantation (FMT) and single bacterial functional experiment, we systematically examine the role of the gut–liver axis in ICA-induced improvement of IUGR-related liver injury, with aim to evaluate the potential of ICA as an early intervention strategy.

## Materials and methods

### Literature survey and visualization

Relevant literature was retrieved from the PubMed database by matching keywords in both titles and abstracts. High-frequency terms were visualized using the PubMedWordcloud package (v0.3.6). Additionally, article titles and abstracts were segmented into single characters, and intersections among character sets were calculated to identify commonly shared terms.

### Molecular docking

The 3D structures of porcine and human PPARα were predicted using AlphaFold3 (https://alphafoldserver.com/) based on amino acid sequences from the UniProt database. The molecular structure of ICA was retrieved from PubChem (https://pubchem.ncbi.nlm.nih.gov/). Docking was performed with AutoDock Vina (The Scripps Research Institute, USA). The conformation with the lowest binding energy was selected and visualized using PyMOL (Schrödinger, LLC, USA), with key interactions (hydrogen bonds, hydrophobic contacts) and binding residues labeled.

### ICA administration to piglets

Thirty-two male piglets (Yorkshire), comprising IUGR (0.87 ± 0.02 kg) and NBW (normal birth weight, 1.61 ± 0.02 kg), were selected and evenly distributed across four lactating Yorkshire sows (*n* = 8 per group). From Day 7 to Day 28, piglets in two of the litters were orally administered ICA (20 mg/kg, Aladdin, I141014). The ICA powder was first dissolved in dimethyl sulfoxide (DMSO), and the solution (10 mg/ml) was then diluted with corn oil (Aladdin, C116023). Each piglet was weighed prior to being gently restrained for precise oral administration of the solution using a sterile syringe to ensure accurate dosing. The dose of 20 mg/kg ICA for piglets was determined by converting a previously reported non-toxic and dose-dependent effective dose of 120 mg/kg in rats [18] using body surface area normalization. Fecal samples were collected weekly from all piglets throughout the experiment. At the end of the trial, piglets were euthanized to collect blood, liver, intestine, and intestinal contents. The preparation of colon content suspension followed previous protocols [19].

### FMT in mice

Four-week-old mice were first treated with a broad-spectrum antibiotic cocktail as per previous studies [19], and fecal samples were collected to confirm the depletion of microbiota. The mice were then randomly divided into three groups (eight mice per group, two cages/group) and received colon content suspension derived from NBW, IUGR, and IUGR + *ICA* piglets. Each mouse was orally gavaged with 200 μl of fecal suspension every other day over a 31-day period and weighed every 3 days.

### *Catenibacterium mitsuokai* administration to mice

Three-week-old male mice were allowed a 5-day acclimation period prior to fecal sample collection. The mice were then randomly divided into four groups (10 mice per group, 2 cages/group) and orally administered phosphate-buffered saline (PBS) buffer (CON), culture liquid (CONL), 3 × 10^8^ CFU/ml of *Catenibacterium mitsuokai* liquid (CmB) or bacterial supernatant (CmS). Each group was gavaged with 200 μl of the corresponding solution every 3 days, with a rest day for body weight measurement, over a 28-day period. During the experiment, all mice had free access to food and water. After euthanasia, relevant samples were collected. Specific pathogen-free (SPF)-grade C57BL/6N mice were purchased from Saiye (Suzhou) Biological Technology Co., Ltd.

### Serum assay and lipid substance measurement

Concentrations of alkaline phosphatase (ALP), alanine aminotransferase (ALT), aspartate aminotransferase (AST), cholinesterase (CHE), total protein (TP), albumin (ALB), globulin (GLOB), triglyceride (TG), total cholesterol (TCHOL), and total bile acid (TBA) in serum were measured using a BX-4000 automatic biochemical analyzer (Sysmex, JPN). The concentration of insulin-like growth factor-1 (IGF-1) in serum was determined using the IMMULITE-2000 fully automated biochemical immunoassay analyzer (Siemens AG, GER). Liver TG and free fatty acid (FFA) levels were quantified using assay kits (Solarbio Science & Technology, BC0620 and BC0595).

### Microbial diversity sequencing and analysis

DNA was extracted from fecal and intestinal contents using the *SteadyPure* Stool DNA Extraction Kit (AGBIO, AG21036). The V3–V4 region of the bacterial 16S rRNA gene was amplified using universal primers 341F and 805R, and polymerase chain reaction (PCR) products were verified by 2% agarose gel electrophoresis, followed by purification with the AxyPrep DNA Gel Extraction Kit (Axygen Biosciences, USA). The purified DNA was quantified using Qubit 3.0 (Life Invitrogen, USA), and library preparation was performed according to Illumina’s genomic DNA library preparation protocol. Finally, paired-end 250 bp sequencing was conducted on the Illumina HiSeq PE250 System (Illumina, USA). The sequencing was conducted by Shanghai BIOZERON Co., Ltd.

Raw sequencing data were processed using Trimmomatic (v0.39) to remove adapter and barcode sequences, and low-quality, unmergeable, and excessively mismatched reads were filtered out to obtain high-quality reads. The DADA2 algorithm in QIIME2 was used to generate amplicon sequence variants (ASVs) [20]. Prior to α and β diversity analyses, samples in each experiment were rarefied to the minimum sequencing depth observed within that experiment to ensure comparability. Taxonomic classification of ASVs was conducted using the uclust algorithm with the SILVA database (SSU138), applying a confidence threshold of 80% to obtain annotation of ASVs [21]. Microbial community analysis was conducted using multiple R packages, where ggalluvial (0.12.5) visualized mean relative abundance at the phylum level, vegan (v2.6-4) computed Bray–Curtis distances and performed non-metric multidimensional scaling (NMDS) analysis (including stress value, Adonis test, and Anosim test significance calculations), ClusterGVis (v2.0.3) generated heatmaps and time-series clustering analyses, MicrobiotaProcess (v1.18.0) facilitated linear discriminant analysis effect size (LEfSe) analysis for identifying differential features and ggtern (v3.3.0) created ternary plots. Microbial co-occurrence networks were constructed using the ggClusterNet package, based on Spearman correlations between ASVs, retaining robust associations with |*r*| > 0.6 and false discovery rate (FDR)-adjusted *P* < .05 (Benjamini–Hochberg correction). Additionally, PICRUSt2 was used for functional predictions of microbial communities.

### Histopathology analysis

Paraffin sections of liver and intestinal tissue were dewaxed, rehydrated, and stained with hematoxylin and eosin (H&E), Alcian Blue, and periodic acid-Schiff solution (AB-PAS). Cryosections were dehydrated with isopropanol, fixed in 4% paraformaldehyde, and then incubated in Oil Red O staining solution. After washing and dehydration, the sections were mounted using Eukitt.

### Scanning and transmission electron microscopy

Samples were fixed in 2.5% glutaraldehyde for 6 h, followed by post-fixation with 1% OsO_4_ for 2 h and rinsed three times with PBS buffer (0.1 M, pH 7.0) for 15 min each. Dehydration was performed through graded ethanol (30%, 50%, 70%, 80%) and acetone (90%, 95%, 100%) solutions. For transmission electron microscopy (TEM), samples were embedded in a mixture of acetone and Spurr resin (1:1 v/v for 1 h, 1:3 v/v for 3 h) and pure Spurr resin overnight, then heated at 65°C for 8 h. Sections were cut using the Leica EM UC7 (Leica, AUT), stained with uranyl acetate and alkaline lead citrate for 5–10 min, and observed under the H-7650 TEM (Hitachi, JPN). For scanning electron microscopy, dehydrated samples were incubated in isoamyl acetate for 1 h, then dried using the HCP-2 critical point dryer (Hitachi, JPN), coated with the Model IB5 ion sputter (Eiko, JPN), and observed under the SU-8010 scanning electron microscope (Hitachi, JPN).

### Immunofluorescence staining

After dewaxing and dehydration, samples were treated with 3% hydrogen peroxide to inhibit endogenous peroxidase activity. Antigen retrieval was performed using EDTA solution (1 mol/l, pH 6.0), followed by permeabilization with normal goat serum (CWBIO, CW0130) for 20 min at 25°C. Samples were then incubated with the MUC2 antibody (Proteintech, 27 675-1-AP) and Goat Anti-Rabbit IgG H&L (Abcam, ab150077), and nuclei were counterstained with 4',6-diamidino-2-phenylindole (DAPI) (CST, #8961) for 5 min.

### Imaging

Images of fluorescence and bright-field were performed on PANNORAMIC MIDI II (3DHISTECH Ltd., HUN). Scanning electron microscopy images were obtained using the SU-8010 (Hitachi, JPN). TEM images were obtained using a Gatan 830 CCD camera (Gatan, USA). Image analysis was performed using CaseViewer (v2.4, 3DHISTECH Ltd., HUN) and ImageJ software (National Institute of Health, USA).

### Enzyme-linked immunosorbent assay (ELISA)

The levels of diamine oxidase (DAO), intestinal trefoil factor (ITF), and LPS were measured using ELISA kits according to the manufacturer’s instructions. Details of all ELISA kits are provided in Table S1.

### Real-time quantitative polymerase chain reaction (RT-qPCR)

Liver tissue was processed according to the instructions of the *SteadyPure* Universal RNA Extraction Kit II (AGBIO, AG21022) to extract RNA. cDNA templates were then synthesized using the *Evo M-MLV* RT Kit with gDNA Clean for qPCR (AGBIO, AG11728). Quantitative real-time PCR (qPCR) was subsequently performed with the SYBR Green Premix *Pro Taq* HS qPCR Kit (AGBIO, AG11701), and amplification and data collection were conducted using a real-time PCR system (Bio-Rad, UK). β-actin served as the internal reference gene, and the relative expression levels of target genes were calculated using the 2^(−∆∆Ct)^ method. For ileal and colonic content samples, the relative abundance of target taxa was assessed with the 16S rRNA gene serving as the internal reference. Primer sequences are provided in Table S1.

### Western blotting

Total protein lysates were extracted using radioimmunoprecipitation assay (RIPA) buffer (Beyotime, P0013B) supplemented with protease and phosphatase inhibitors (Beyotime, P1010; P1050). Protein concentration was measured with the BCA Protein Assay Kit (Beyotime, P0010S). Equal amounts of protein were separated by 10% polyacrylamide gel electrophoresis and transferred onto polyvinylidene fluoride membranes. After blocking with QuickBlock Blocking Buffer for Western Blotting (Beyotime, P0252), the membranes were incubated overnight at 4°C with primary antibodies. Based on the molecular weight marker, the gel was sectioned to include target proteins of different molecular weights along with the internal reference protein, GAPDH. For proteins with similar molecular weights, membranes were stripped using stripping buffer (NCM Biotech, WB6500) before incubation with another primary antibody. Secondary antibody incubation was carried out at room temperature for 1 h. Signals were detected using BeyoECL Star (Beyotime, P0018AS), and protein bands were visualized with a gel documentation system (Thermo Fisher Scientific, Waltham, MA). Densitometric analysis was performed using ImageJ software. Antibodies are listed in Table S1.

### Antibacterial assay by the paper disc method

The *C. mitsuokai* strain was obtained from the German Collection of Microorganisms and Cell Cultures (DSM 15897). The liquid culture medium was prepared as follows: 57.5 g of minced meat carbohydrate broth base (Mingzhoubio, KDM150) was added to 1 l of deionized water, heated to a boil, and kept boiling for more than 1 min. The solution was then dispensed into test tubes. A suitable amount of minced beef particles (Mingzhoubio, KDM150-1), approximately one-third of the liquid volume, was added to each tube, followed by autoclaving at 121°C for 30 min and cooling below 50°C. Under sterile conditions, 0.5 mg of hemin chloride (Mingzhoubio, KDM152) and 5 mg of vitamin K1 (Mingzhoubio, KDM153) were added per 100 ml of the medium. The medium was then placed in the AW200SG anaerobic workstation (ELECTROTEK, UK) for 48 h of deoxygenation before being used to culture *C. mitsuokai*.

For the assay, 200 μl of the bacterial suspension was evenly spread on a Columbia blood agar plate to ensure an even distribution. Filter paper disks (blank, 50 μl ICA solvent [DMSO and 10% Tween 20, 1:2], and 50 μl of gradient concentrations of ICA solution) were gently placed on the inoculated plate surface, ensuring full contact between the paper and the agar surface. Finally, the plate was incubated under anaerobic conditions at 37°C for 24 h to observe the formation of inhibition zones.

### Construction of standard plasmids and absolute quantification

Specific 141 bp fragments were amplified from the *C. mitsuokai* genome using 2× Phanta Max Master Mix (Vazyme, P515). The amplification products were analyzed by 1% agarose gel electrophoresis and subjected to gel extraction. To construct the standard plasmid, the pCS2 vector was linearized and digested with DpnI enzyme (ABclonal, RK21109) to remove methylated DNA templates, followed by gel extraction. The products were then ligated using 2× MultiF Seamless Assembly Mix (ABclonal, RK21020) via homologous recombination. The ligated product was transformed into DH5α competent *Escherichia coli* strain (42°C, 45 s) and plated on LB agar plates. Two positive clones were selected for liquid culture, followed by Sanger sequencing. Primer sequences are provided in Table S1.

Following the construction of the standard plasmids, plasmid DNA was extracted, and the copy number was calculated based on concentration and base length. After determining the optimal dilution factor, RT-qPCR was performed to generate a standard curve. The cycle threshold (CT) values from the samples were used to calculate the copy number of *C. mitsuokai*, enabling absolute quantification analysis based on the weight.

### Prokaryotic reference transcriptome sequencing and analysis

The 24 mg/ml ICA corresponding to the maximum inhibition zone was selected. To prepare the treatment mixture, 1 ml of PBS buffer, 1 ml of ICA solution, and 1 ml of a mixture containing DMSO and 10% Tween 20 (1:2) were added to the liquid culture medium, ensuring that the final DMSO concentration was below 0.05%. Each treatment was conducted with three replicates. Then, 200 μl of *C. mitsuokai* culture was added and incubated for 12 h. After incubation, the culture was centrifuged at 6000 × *g* for 5 min, and the supernatant was discarded, while the pellet was retained for RNA extraction.

Total RNA was extracted from the samples using TRIzol reagent (Invitrogen, USA), and rRNA was depleted using the Illumina Ribo-Zero Plus rRNA Depletion Kit (Illumina, USA). After purifying Poly(A) RNA, reverse transcription, and amplification, the cDNA library was constructed with the TruSeq RNA Library Prep Kit v2 (Illumina, USA) and sequenced using paired-end 150 bp on the Illumina Novaseq 6000 System (Illumina, USA). The sequencing was conducted by Shanghai BIOZERON Co., Ltd.

Raw fastq data were processed with fastp software (v0.23.1), removing adapter contamination, low-quality bases, and ambiguous bases to obtain high-quality reads. Valid reads were then aligned with the Rfam database using BLAST+ (v2.7.1) with an E-value threshold of ≤1e − 5 to eliminate rRNA sequences. The remaining valid reads were mapped to the ASM2514878v1 reference genome using Rockhopper [22] software (v2.0.3), generating a matrix of reads per kilobase of transcript per million mapped reads (RPKM) values. Differential gene expression analysis was performed using the DESeq package (v1.38.3) with default settings based on a negative binomial distribution model, considering genes with FDR-adjusted *P* (Benjamini–Hochberg correction) < 0.05 and |log2 FoldChange| > 1 as significantly differentially expressed. KEGG pathway enrichment analysis was conducted using the clusterProfiler package (v4.0). Hierarchical clustering and principal component analysis (PCA) were performed using the stats package (v4.1.5), and gene co-expression modules were constructed using the WGCNA package (v1.72-1) with the following parameters: soft-threshold power was set to 8, minimum module size was 30, modules were merged at a height cut-off of 0.25, and an unsigned topology was used to build the network.

### Statistical analysis

High-throughput data were analyzed statistically using the appropriate R packages. Significant differences were assessed using the Mann–Whitney U test. Data from at least six independent experiments were presented as means ± standard error of the mean or median values with IQR. All statistical analyses and graph generation were conducted in R (v4.1.2).

## Results

### Suckling-period ICA intervention mitigates disruption of hepatic PPAR signaling by reducing LPS translocation

Using the three-core keyword, “PPARα agonist,” “NF-κB inhibitor,” and “hypoxia mitigation,” a total of 837, 1146, and 811 relevant publications were identified, respectively. By extracting key terms from the titles of these articles and conducting intersection analysis, ICA emerged as a potential multi-target therapeutic candidate capable (Fig. S1A). Molecular docking analysis was conducted to further investigate the interaction between ICA and PPARα. The results demonstrated that ICA binds stably to human PPARα, with a binding energy of −8.1 kcal/mol (Fig. S1B). Similarly, ICA exhibited a binding energy of −7.3 kcal/mol with porcine PPARα, forming multiple hydrogen bonds and hydrophobic interactions with several amino acid residues (Fig. S1C). Further analysis of the mRNA levels of PPAR signaling pathway-related genes in liver, which was performed after the end of the ICA intervention period (Day 28), showed that ICA significantly corrected the abnormal expression of lipid metabolism-related genes in IUGR piglets (Fig. S2A–D). Compared to IUGR piglets, IUGR + *ICA* piglets exhibited a significant increase in *PPARA* mRNA levels and a significant decrease in *PPARG* and *TLR4* mRNA levels. The mRNA levels of the lipid droplet marker *PLIN2* and the lipid transport-related *APOA4* were reduced. And the mRNA level of the lipolysis-related *SCD* was significantly increased, whereas the mRNA levels of fatty acid transport-related *ACSL1* and fatty acid oxidation-related *ACADL*, *ACOX1*, and *CPT1A* were significantly decreased in the IUGR + *ICA* piglets than in IUGR piglets. The mRNA levels of *ANGPTL4*, *APOA1*, *ME1*, and *CD36* were significantly increased in IUGR piglets than in the NBW piglets; however, these levels did not change significantly after ICA treatment. In contrast, the mRNA levels of *LPL*, *SLC27A4*, *ACOX3*, and *EHHADH* were significantly higher in both IUGR and IUGR + *ICA* piglets than in NBW piglets.

Evaluation of intestinal barrier function revealed that serum LPS levels were significantly higher in IUGR piglets than in NBW piglets at Day 7 (Fig. S2E). By Day 28, both IUGR and IUGR + *ICA* piglets exhibited significantly elevated LPS levels compared to NBW piglets; however, LPS levels in IUGR + *ICA* piglets were significantly lower than those in IUGR piglets (Fig. S2F). Similarly by Day 28, the level of DAO was significantly higher whereas the level of ITF was significantly lower in both IUGR and IUGR + *ICA* piglets than in the NBW piglets, however, IUGR + *ICA* piglets showed significantly decrease in the level of DAO and significantly increase in the level of ITF compared to IUGR piglets (Fig. S2G).

### Suckling-period ICA intervention improves intestinal morphology and mucosal barrier

H&E staining and scanning electron microscopy images of the ileum revealed that (Fig. S3A), compared to NBW piglets, which exhibited clear, intact villi, NBW + *ICA* and IUGR piglets showed significant atrophy and even thickening, with sparse and disorganized arrangements in ileal villi. In comparison to IUGR piglets, IUGR + *ICA* piglets exhibited some villus shedding but had a clearer and more intact overall villus structure, with tightly arranged and nearly normal morphology. The villi ultrastructure appeared more regular and cylindrical, with only a few missing microvilli, and the overall arrangement was restored to an orderly state.

H&E staining and TEM images of the colon revealed that (Fig. S3B), compared to NBW piglets, whose colonic glands were regularly arranged, NBW + *ICA* and IUGR piglets exhibited structural abnormalities in the colonic glands. Compared to IUGR piglets, IUGR + *ICA* piglets still showed some immune cell infiltration in certain areas; however, their glandular structure had improved. The arrangement was more regular, with intact and continuous epithelium, neat edges, and a morphology approaching normal. The microvilli were more tightly and orderly arranged, with continuous and clearly visible intercellular junctions such as tight junctions and desmosomes.

AB-PAS and MUC2 fluorescence staining revealed a significant reduction in staining intensity in the ileum and colon of IUGR piglets. Specifically, the goblet cell-derived mucin content represented by AB-PAS signal was completely absent in some glands, and the positive signal of MUC2 showed an irregular, focal distribution. In contrast, IUGR + *ICA* piglets displayed stronger and more continuous staining in both the ileum and colon, a litter weaker than but nearly to the NBW piglets (Fig. S3C and D).

### Suckling-period ICA intervention remodels the dynamic trajectory of the gut microbiota

**Figure 1:**
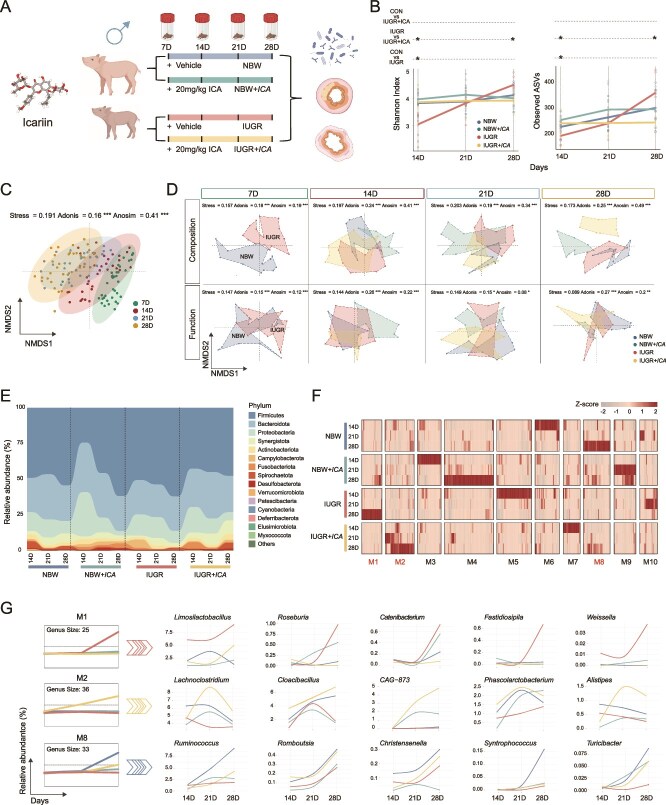
Dynamic changes of ICA intervention on the fecal microbiota of male IUGR piglets. (A) Schematic diagram of the experimental design. (B) Changes in α-diversity. (C) Age-related variations in β-diversity. (D) Changes of β-diversity in microbial composition and function. (E) Changes in microbial composition at the phylum level. (F) Temporal clustering at the genus level. (G) Specific up-regulated module at the genus level. ICA, icariin; IUGR, intrauterine growth restriction. Statistical significance was determined using the Mann–Whitney U test (B) (^*^P < .05).

By collecting fecal samples from male piglets at 7, 14, 21, and 28 days of age, we uncovered the dynamic changes in gut microbiota during ICA intervention (Fig. 1A). Alpha diversity analysis revealed that at 14 days of age, the Shannon Index and Observed ASVs were significantly lower in IUGR piglets compared to NBW piglets. In IUGR + *ICA* piglets, the Shannon Index was significantly higher at Day 14, whereas the Observed ASVs showed a significant increase at Day 14 and a decrease at Day 28 compared to IUGR piglets (Fig. 1B). Beta diversity analysis demonstrated a distinct temporal distribution pattern (Fig. 1C), with IUGR piglets exhibiting significant differences from NBW piglets throughout the study period. As ICA intervention progresses, the microbial composition of NBW *+ ICA* and IUGR *+ ICA* piglets gradually diverged from that of NBW and IUGR piglets, respectively (Fig. 1D).

At the phylum level, NBW piglets maintained a relatively stable microbial composition, whereas IUGR piglets exhibited pronounced fluctuations, particularly in the abundances of *Firmicutes* and *Bacteroidota*, which significantly differed from those in NBW piglets. ICA treatment induced marked alterations in the microbial composition of both NBW and IUGR piglets, with significant shifts in the abundances of *Firmicutes*, *Bacteroidota*, and *Proteobacteria*, particularly at Day 14. Additionally, compared to IUGR piglets, IUGR + *ICA* piglets exhibited notable differences in the abundances of *Firmicutes* and *Bacteroidota* (Fig. 1E).

At the genus level, the temporal dynamics of fecal microbiota revealed 10 distinct modules (Fig. 1F and G). In NBW piglets, genera that increased over time were primarily clustered in module M8 (e.g. *Ruminococcus*, *Romboutsia*, *Christensenella*, *Syntrophococcus*, *Turicibacter*), whereas those decreased genera were grouped in module M6. In NBW *+ ICA* piglets, increasing genera were assigned to module M4, and decreasing genera clustered in module M3. IUGR piglets exhibited an increase in genera within module M1 (e.g. *Limosilactobacillus*, *Roseburia*, *Catenibacterium*, *Fastidiosiplla*, *Weissella*), and declining genera were categorized into module C5. In IUGR + *ICA* piglets, genera that increased over time were grouped into module M2 (e.g. *Lachnoclostridium*, *Cloacibacillus*, *CAG-873*, *Phascolarctobacterium*, *Alistipes*), whereas those that decreased were classified into module C7. And genera in modules M8 and M1 were more abundant in NBW and IUGR *+ ICA* piglets than in IUGR piglets (Fig. 1G).

### Suckling-period ICA intervention refines the intestinal microbiota community to reduce LPS production

Microbial community analysis of ileal contents revealed a significant increase in the Observed ASVs in IUGR + *ICA* piglets compared to NBW + *ICA* and IUGR piglets, whereas the Shannon Index showed no significant differences among groups (Fig. S4A). ICA treatment led to distinct shifts in microbial community structure and function in both NBW and IUGR piglets (Fig. S4B), with notable changes in the abundance of *Proteobacteria* (Fig. S4C). Network analysis (Fig. S4D) showed that in NBW piglets, core ASVs were primarily *Ligilactobacillus*, with *Lactobacillus* and *Terrisporobacter* as key connectors. IUGR piglets exhibited the sparsest network, with core ASVs mainly comprising *Lactobacillus*, *Treponema*, and *Clostridium sensu stricto*, connected through *Streptococcus*. The most enriched network was observed in IUGR *+ ICA* piglets, where core ASVs consisted of *Lactobacillus*, *Romboutsia*, and *Clostridium sensu stricto*, with *Terrisporobacter* as a key connector. Furthermore, network connectivity was significantly higher in IUGR + *ICA* piglets than in other groups upon node removal (Fig. S4E). LEfSe analysis identified distinct microbial enrichments across groups (Fig. S4F). Ternary analysis further illustrated microbial distribution patterns (Fig. S4G), showing that NBW and IUGR + *ICA* piglets had more unique genera. ICA treatment restored the relative abundances of *Terrisporobacter* and *Romboutsia* in IUGR piglets similar to NBW piglets. Further clustering analysis of high-expression KEGG Orthology (KOs) predicted from *Romboutsia* and *Terrisporobacter* ASV sequences revealed that KOs reduced in IUGR piglets but increased in IUGR + *ICA* piglets were strongly linked to quorum sensing, bacterial chemotaxis, and fatty acid degradation pathways (Fig. S4H). RT-qPCR confirmed that the abundances of *Terrisporobacter* and *Romboutsia* were significantly lower in IUGR piglets than in NBW piglets, however, significantly higher in IUGR + *ICA* piglets than in IUGR piglets (Fig. S4I).

**Figure 2:**
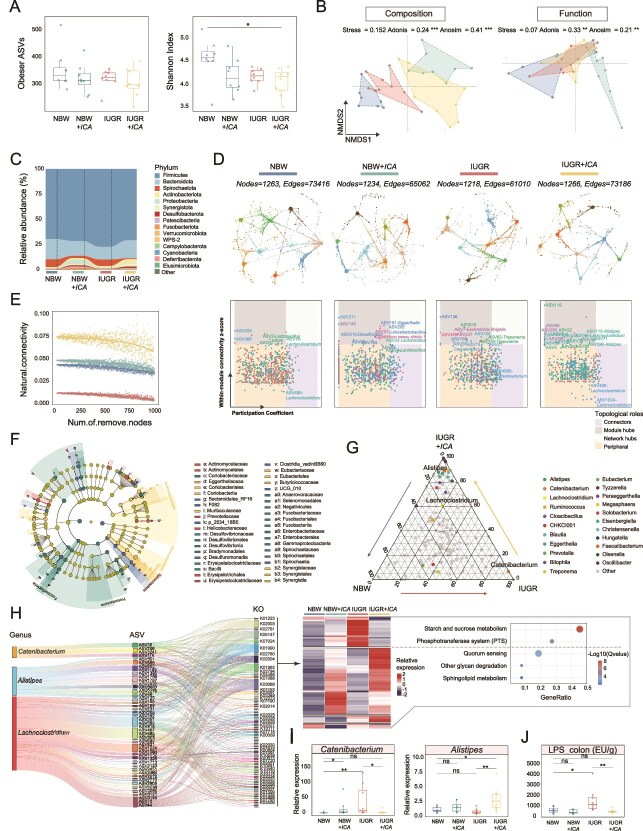
Changes of ICA intervention on the colonic microbiota of male IUGR piglets. (A) Changes in α-diversity. (B) Changes of β-diversity in microbial composition and function. (C) Changes in microbial composition at the phylum level. (D) Changes in the microbial network. (E) Stability of microbial networks. (F) LEfSe analysis of different microbiota. (G) Ternary analysis of different genera. (H) Functional prediction and enrichment analysis of crucial genera. (I) Changes in relative abundance of *Catenibacterium* and *Alistipes* using RT-qPCR. (J) The level of LPS in colonic contents. ICA, icariin; IUGR, intrauterine growth restriction. Statistical significance was determined using the Mann–Whitney U test (A, I, J) (^**^P < .01; ^*^P < .05; ns *P* > .05).

Microbial community analysis of colonic contents revealed a significantly lower Shannon Index in IUGR + *ICA* piglets compared to NBW piglets, whereas the Observed ASVs showed no significant differences among groups (Fig. 2A). The colonic microbial community structure exhibited distinct distribution patterns across groups (Fig. 2B). IUGR piglets had a higher abundance of *Firmicutes* and a lower abundance of *Bacteroidota*. ICA treatment significantly reduced the abundance of *Spirochaetota* in both NBW and IUGR piglets (Fig. 2C). Network analysis (Fig. 2D) showed that core ASVs were primarily composed of *Lactobacillus*, with *Lachnoclostridium* as the network hub in NBW piglets. In NBW *+ ICA* piglets, core ASVs mainly included *Eggerthella*, *Desulfovibrio*, and *Clostridium sensu stricto*, with *Lactobacillus* as the key connector. IUGR piglets exhibited the sparsest network, dominated by *Escherichia-Shigella*, *Treponema*, and *Catenibacterium*, with *Catenibacterium* as the network connector. The most enriched network was observed in IUGR + *ICA* piglets, where *Lachnoclostridium* and *Alistipes* were the core ASVs, with *Lachnoclostridium* as the central hub. Moreover, upon node removal, the network connectivity of the colonic microbiota was significantly higher in IUGR + *ICA* piglets than in IUGR piglets (Fig. 2E). LEfSe analysis identified distinct microbial enrichments across groups (Fig. 2F). Ternary analysis further revealed that IUGR + *ICA* piglets had more unique genera, and IUGR piglets exhibited a high abundance of *Catenibacterium* (Fig. 2G). Additionally, ICA treatment significantly increased the relative abundances of *Alistipes* and *Lachnoclostridium* in IUGR piglets. Clustering analysis of highly expressed KOs predicted from ASV sequences of *Catenibacterium* revealed that in IUGR piglets, significantly upregulated KOs were strongly associated with starch and sucrose metabolism pathways. In contrast, in IUGR + *ICA* piglets, upregulated KOs were highly correlated with quorum sensing (Fig. 2H). RT-qPCR further confirmed that the abundance of *Alistipes* was significantly higher in IUGR + *ICA* piglets than in NBW and IUGR piglets, whereas the abundance of *Catenibacterium* was significantly lower in IUGR + *ICA* piglets than in IUGR piglets (Fig. 2I). Additionally, the level of LPS in the colonic contents of IUGR piglets was significantly higher than that in NBW piglets, whereas no significant difference was found between IUGR + *ICA* piglets and NBW piglets, and the level of LPS was significantly lower in IUGR + *ICA* piglets than in the IUGR piglets (Fig. 2J).

### FMT replicates the IUGR piglet phenotype and verify the outcomes of ICA intervention

**Figure 3:**
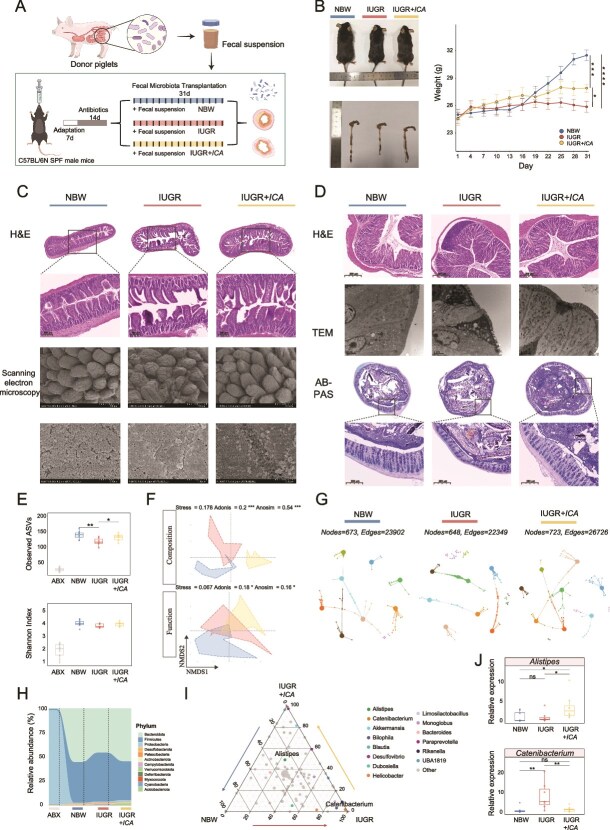
The effect of microbiota transplantation from piglets on intestinal permeability and microbiota composition. (A) Schematic diagram of the experimental design. (B) The temporal change in body weight. (C) H&E staining and scanning electron microscopy images of the ileum. (D) H&E staining, TEM images, and AB-PAS staining of the colon. (E) Changes in α-diversity. (F) Changes of β-diversity in microbial composition and function. (G) Changes in the microbial network. (H) Changes in microbial composition at the phylum level. (I) Ternary analysis of different genera. (J) Changes in relative abundance of *Catenibacterium* and *Alistipes* using RT-qPCR. TEM, transmission electron microscopy. Statistical significance was determined using the Mann–Whitney U test (E, J) (^****^P < .0001; ^***^P < .001; ^**^P < .01; ^*^P < .05; ns *P* > .05).

To investigate the role of gut microbiota in mediating the regulatory effects of ICA on hepatic lipid metabolism in male IUGR piglets, we conducted an FMT experiment (Fig. 3A). Colonic content from each donor piglet (NBW, IUGR, or IUGR + *ICA*) was transferred to a single mouse, without any additional selection or exclusion criteria applied to donors. After 4 weeks of oral administration of piglet-derived fecal microbiota, both IUGR and IUGR + *ICA* mice exhibited significantly lower body weights than NBW mice. However, IUGR + *ICA* mice showed a significant increase in body weight compared to IUGR mice (Fig. 3B). The morphology and ultrastructure of the ileum and colon in recipient mice closely resembled the donor phenotype (Fig. 3C and D).

Antibiotic treatment led to a reduction in the observed ASVs and Shannon Index of fecal microbiota across all mice. Following 4 weeks of piglet-derived fecal microbiota administration, the observed ASVs of colonic microbiota was significantly lower in IUGR mice than in NBW mice, but significantly higher in IUGR + *ICA* mice compared to IUGR mice (Fig. 3E). The structure and function of the colonic microbiota exhibited notable differences among the three groups (Fig. 3F). Network analysis revealed that FMT mice successfully recapitulated the colonic microbiota characteristics observed in piglets. The microbial network in IUGR mice was the sparsest, whereas the network in IUGR + *ICA* mice was the most complex (Fig. 3G). After 4 weeks of piglet-derived FMT, IUGR mice exhibited a higher abundance of *Firmicutes* and a lower abundance of *Bacteroidota* in the colonic microbiota (Fig. 3H). Further ternary analysis and RT-qPCR confirmed that *Catenibacterium* was significantly enriched in IUGR mice, whereas *Alistipes* was significantly enriched in IUGR + *ICA* mice (Fig. 3I and J).

**Figure 4:**
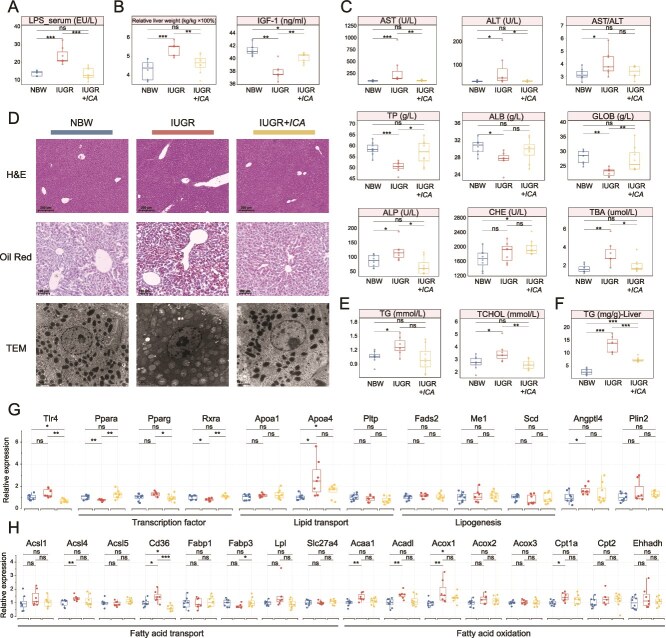
The effect of microbiota transplantation from piglets on hepatic lipid metabolism. (A) The level of LPS in serum. (B) Changes in relative liver weight and the level of IGF-1 in serum. (C) Liver injury-related and synthesis-related serum indicators. (D) H&E and oil red O staining and TEM images of the liver. (E) The levels of TG and TCHOL in serum. (F) The levels of TG in liver. (G) Alterations in mRNA expression of *TLR4*, transcription factor, lipid transport, lipogenesis-related genes and lipid droplet marker genes. (H) Alterations in mRNA expression of fatty acid transport and oxidation-related genes. LPS, lipopolysaccharide; AST, aspartate aminotransferase; ALT, alanine aminotransferase; TP, total protein; ALB, albumin; GLOB, globulin; ALP, alkaline phosphatase; CHE, cholinesterase; TBA, total bile acid; TG, triglyceride; TCHOL, total cholesterol; IGF-1, insulin-like growth factor-1. Statistical significance was determined using the Mann–Whitney U test (A–C, E–H) (^****^P < .0001; ^***^P < .001; ^**^P < .01; ^*^P < .05; ns *P* > .05).

Compared to other recipients, IUGR mice had significantly higher level of serum LPS (Fig. 4A), along with an increased liver index and a reduction in the level of serum IGF-1 (Fig. 4B). In terms of serum liver function, the levels of AST, ALT, ALP, TBA, as well as the AST/ALT ratio were significantly elevated in IUGR mice than those in the NBW mice. Meanwhile, the levels of TP, ALB, and GLOB were markedly decreased in IUGR mice than in NBW mice. In contrast, the level of serum CHE in IUGR + *ICA* mice was significantly increased, with no notable differences in other indicators (Fig. 4C). Distinct liver injury characteristics in IUGR mice were revealed by H&E staining and TEM image, including extensive lipid droplet accumulation in hepatocytes, vacuolar degeneration, hepatocyte swelling, and necrosis in certain regions, an increased level of lipid droplets of varying sizes, mitochondrial swelling, and cristae disappearance. In contrast, no significant pathological changes were observed in IUGR + *ICA* mice (Fig. 4D). Further analysis of lipid-related substances indicated that the levels of TG and TCHOL were significantly higher, along with an increased level of hepatic TG in the serum of IUGR mice than those in the NBW mice. However, the level of hepatic TG remained significantly elevated in IUGR + *ICA* mice than in the NBW mice, whereas it was lower than that in IUGR mice (Fig. 4E and F). Analysis of PPAR signaling pathway-related genes in liver samples revealed that, compared to those in the IUGR mice, the levels of *Ppara*, *Rxra*, and *Fabp3* mRNA in IUGR + *ICA* mice were significantly higher, and the levels of *Tlr4*, *Pparg*, and *Cd36* mRNA were significantly lower. Additionally, the levels of *Angptl4*, *Acsl4*, *Acaa1*, *Acadl*, *Acox1*, and *Cpt1a* mRNA in IUGR mice were markedly elevated compared to those in the NBW mice. However, no significant differences in the expression of these genes were observed in IUGR + *ICA* mice (Fig. 4G and H).

### Oral administration of *C. mitsuokai* induces a gut–liver axis response resembling IUGR

**Figure 5:**
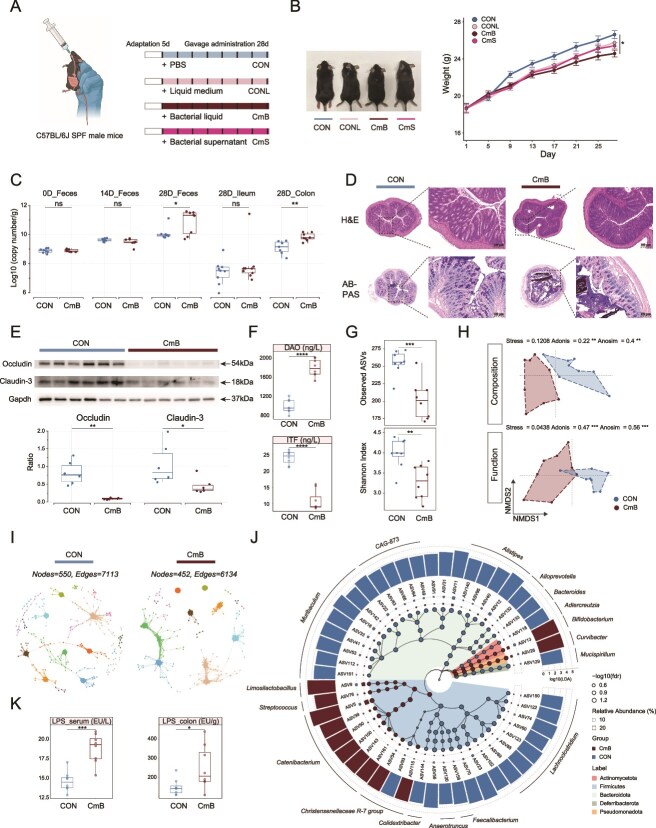
The effect of oral administration of *C. mitsuokai* on colonic permeability and microbiota composition. (A) Schematic diagram of the experimental design. (B) The temporal change in body weight. (C) Absolute quantification of *C. mitsuokai* across multiple time points and intestinal segments. (D) H&E and AB-PAS staining of the colon. (E) Changes in the protein levels of occludin and claudin-3. (F) The levels of DAO and ITF in serum. (G) Changes in α-diversity. (H) Changes in β-diversity of microbial composition and function. (I) Changes in the microbial network. (J) LEfSe analysis of different microbiota. (K) The level of LPS in serum and colonic contents. DAO, diamine oxidase; ITF, intestinal trefoil factor; LPS, lipopolysaccharide; IUGR, intrauterine growth restriction. Statistical significance was determined using the Mann–Whitney U test (B, C, E–G, K) (^****^P < .0001; ^***^P < .001; ^**^P < .01; ^*^P < .05; ns *P* > .05).

To investigate the function of *C. mitsuokai*, we performed gavage treatments on mice with live CmB or its CmS with blank CONL and CON as controls (Fig. 5A). After 4 weeks of treatment, CmB mice showed a significant reduction in body weight compared to CON mice (Fig. 5B). We successfully constructed a standard plasmid for *C. mitsuokai* and the corresponding reliable standard curve (*R*^2^ = 0.99, Fig. S5A and B). Absolute quantitative analysis revealed no significant difference in the copy number of *C. mitsuokai* in the feces of CmB mice on Day 0 and Day 14 compared to CON mice, but a significant increase was observed on Day 28. Additionally, on Day 28, no significant differences were found in bacterial number in the ileal contents between CmB and CON mice, whereas a significant increase was noted in the colon contents (Fig. 5C). Histological examination of the colon using H&E and AB-PAS staining showed incomplete epithelial borders in the CmB mice, with abnormal glandular structures, irregular crypt depth and diameter, immune cell infiltration, reduced goblet cell numbers, and diminished mucus staining signals (Fig. 5D). Compared to CON mice, protein levels of tight junction proteins like occludin and claudin-3 were significantly reduced in the colon of CmB mice (Fig. 5E), in serum, the level of DAO was significantly elevated and the level of ITF was significantly decreased (Fig. 5F).

**Figure 6:**
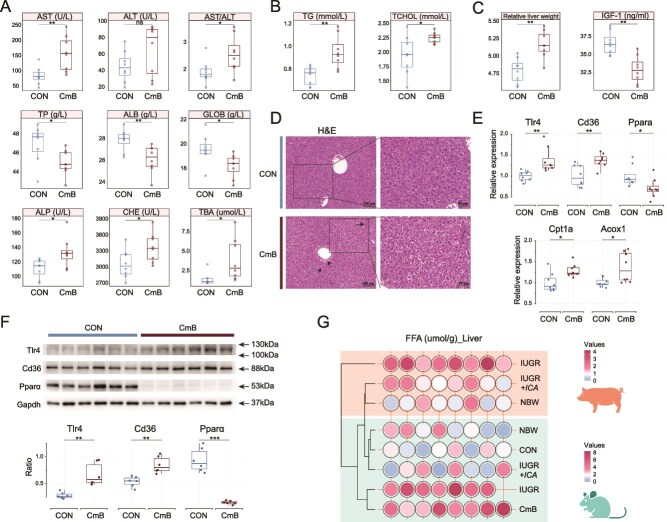
The effect of oral administration of *C. mitsuokai* on hepatic lipid metabolism. (A) Liver injury-related and synthesis-related serum indicators. (B) The levels of TG and TCHOL in serum. (C) Changes in relative liver weight and the level of IGF-1 in serum. (D) H&E staining of the liver. (E) Alterations in mRNA expression of *Tlr4*, *Cd36*, *Pparα*, *Cpt1a,* and *Acox1*. (F) Changes in the protein levels of Tlr4, Cd36, and Pparα. (G) The levels of FFA in serum across all experiments. AST, aspartate aminotransferase; ALT, alanine aminotransferase; TP, total protein; ALB, albumin; GLOB, globulin; ALP, alkaline phosphatase; CHE, cholinesterase; TBA, total bile acid; TG, triglyceride; TCHOL, total cholesterol; IGF-1, insulin-like growth factor-1; FFA, free fatty acid. Statistical significance was determined using the Mann–Whitney U test (A–C, E, F) (^***^P < .001; ^**^P < .01; ^*^P < .05; ns *P* > .05).

Further microbiota sequencing analysis revealed that observed ASVs and Shannon Index of the colon microbiota in CmB mice were significantly lower than those in CON mice (Fig. 5G), with significant differences in the structure and functional distribution of the microbiota (Fig. 5H). The microbial association network in the colon of CmB mice was sparse, containing 452 nodes and 6134 edges (Fig. 6I). Following gavage with live *C. mitsuokai*, the composition of *Firmicutes* and *Bacteroidota* in the colon microbiota underwent significant changes. Specifically, the abundances of genera such as *Catenibacterium*, *Limosilactobacillus*, and *Streptococcus* increased, and the abundances of genera like *CAG-873*, *Lachnoclostridium*, *Alistipes*, and *Muribaculum* decreased (Fig. 5I). Besides, the level of LPS was significantly elevated in both serum and colon contents of CmB mice compared to that in the CON mice (Fig. 5K).

In terms of serum liver function, compared to CON mice, the CmB mice had significantly higher levels of AST, ALP, CHE, TBA, TG, TCHOL, and the AST/ALT ratio, and significantly lower levels of TP, ALB, and GLOB (Fig. 6A and B). And the liver index of CmB mice was significantly increased, and the level of IGF-1 in serum was significantly reduced (Fig. 6C). H&E staining of the liver in CmB mice revealed disorganized hepatocyte arrangement, widened intercellular spaces, and vacuolization, along with sparse immune cell infiltration (Fig. 6D). Compared to CON mice, the CmB mice had significantly higher mRNA levels of *Tlr4*, *Cd36*, *Cpt1a*, and *Acox1*, and significantly lower mRNA level of *Ppara* in the liver (Fig. 6E). Furthermore, the protein levels of Tlr4 and Cd36 significantly increased, and the protein level of Pparα significantly decreased in CmB mice (Fig. 6F). The integrated analysis showed that, despite interspecies differences, the abnormalities in the level of FFA were clearly observed in liver from IUGR piglets, mice receiving fecal microbiota from IUGR piglets, and CmB mice, along with the regulatory effect of ICA intervention on these abnormalities (Fig. 6G).

### The potential mechanism of ICA-mediated inhibition on the growth of *C. mitsuokai*

**Figure 7:**
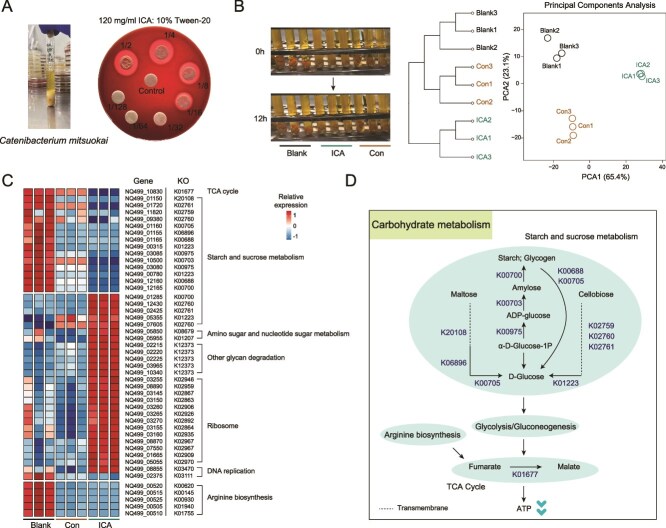
The impact of ICA on the growth of *C. mitsuokai*. (A) The antibacterial activity of ICA against *C. mitsuokai*. (B) Transcriptional profiles within *C. mitsuokai* after treated with ICA for 12 h. (C) Differential genes and functional annotations. (D) Schematic diagram of carbohydrate metabolism. ICA, icariin.

The antibacterial activity of ICA against *C. mitsuokai* was assessed using the filter paper disc method. The results demonstrated that ICA effectively inhibited the growth of *C. mitsuokai* (Fig. 7A). The mRNA expression profiles of *C. mitsuokai* were compared after 12 h under different treatment conditions, which included: (i) untreated group (Blank), (ii) ICA solvent-treated group (DMSO + 10% Tween 20, Con), and (iii) ICA-treated group (1:4, 24 mg/ml, ICA). The expression matrix of RPKM values are presented in Table S2. Cluster analysis and PCA of the expression matrix indicated that samples from the Blank and Con groups clustered together in the phylogenetic tree, with no clear separation along the axis of PCA1. In contrast, samples from ICA group formed a distinct cluster and were significantly separated from the other groups along the axis of PCA1 (Fig. 7B). Further differential gene analysis revealed that, compared to the Blank group, functional genes related to the arginine biosynthesis pathway were significantly downregulated in both the Con and ICA groups. Moreover, the ICA group exhibited significant alterations in genes associated with carbohydrate metabolism, ribosome function, and DNA replication (Fig. 7C). The substantial downregulation of genes related to starch and sucrose metabolism may impact ATP synthesis (Fig. 7D).

## Discussion

The gut microbiota is essential for maintaining host health and metabolism. Longitudinal analysis of piglet fecal samples from birth to weaning revealed significant age-dependent shifts in microbiota composition, consistent with previous findings [23]. The fecal microbiota in IUGR piglets at Day 7, exhibited distinct compositional and functional differences compared to the normal piglets, further supporting the notion that IUGR piglets develop a unique gut microbiota profile early in life [24]. Nonetheless, following ICA intervention, fecal microbial richness and diversity of IUGR piglets closely resembled those of normal piglets. Further clustering analysis of genus abundance revealed that *Firmicutes* predominated in the gut microbiota of IUGR piglets, with elevated levels of *Limosilactobacillus* and *Roseburia*, consistent with previous studies [14]. The distinction is that a significant reduction in the abundance of *Catenibacterium* was observed in IUGR piglets after ICA intervention. Previous studies have shown that the abundance of *Catenibacterium* is negatively correlated with neonatal weight, fetal biparietal diameter, head circumference, and femur length [25].

The microbiota composition varies across different regions of the gastrointestinal tract. Previous studies have shown that both the structure and function of the small and large intestines in IUGR piglets are impaired [26], resulting in lower microbial diversity and a distinct microbiota composition compared to normal piglets [13, 14]. In our study, ICA intervention significantly enhanced microbial diversity in the ileum of IUGR piglets, with a marked restoration in the abundance of *Terrisporobacter* and *Romboutsia* genera. These genera are known to play a positive role in maintaining gut health and homeostasis [27, 28]. In terms of the colonic microbiota, ICA intervention did not significantly alter its diversity, but it effectively reshaped the microbiota composition. This was evidenced by a reduction in the abundance of *Firmicutes* and *Spirochaetota* phyla, along with an increase in *Bacteroidota* phylum. The abundance of *Lachnoclostridium*, *Catenibacterium*, and *Alistipes* genera exhibited notable changes, aligning with dynamic shifts in fecal microbiota and indicating a closer resemblance between the colon and fecal microbiota composition [29]. Furthermore, *Catenibacterium* is closely associated with energy metabolism [30], with functional predictions showing its enrichment in starch and sucrose metabolism pathways, reflecting an imbalance in the energy metabolism homeostasis within the intestines of IUGR piglets.

The colonization, succession, and balance of the gut microbiota directly influence intestinal barrier function [31]. In our study, after ICA intervention, normal piglets exhibited morphological changes and hyperplastic responses in the ileum and colon due to drug stimulation, which was consistent with the previously observed ICA-induced liver injury [2]. However, for IUGR piglets, ICA intervention markedly enhanced the integrity of ileal and colonic structures. The structural integrity of villi and crypts forms the fundamental basis of intestinal barrier function [32]. Consequently, ICA intervention in IUGR piglets led to decreased levels of DAO and LPS, increased levels of ITF in the serum, and enhanced MUC2 signal intensity in the ileum and colon, collectively indicating a significant improvement in intestinal barrier function. Previous studies have confirmed that intestinal barrier damage facilitates LPS translocation into the portal circulation, contributing to various liver diseases [33]. We found that ICA intervention not only reshaped the colonic microbiota composition in IUGR piglets but also led to a reduction in LPS production. Mice receiving fecal transplantation from IUGR piglets exhibited pathological changes, including focal hepatocellular necrosis and significant lipid accumulation, accompanied by a compensatory and protective response characterized by the upregulated expression of fatty acid β-oxidation-related genes, such as *Cpt1a* and *Acox1* [34], as well as Apoa4 [2]. In contrast, mice transplanted with feces from ICA-treated IUGR piglets exhibited effective activation of hepatic *Pparα*, along with decreased expression of *Tlr4* and *Cd36*, further regulating lipid metabolism-related gene expression and significantly alleviating lipid deposition and liver damage. Although the recipient mice did not fully retain the same microbial community as the donor piglets, the differences in their colonic microbiota composition closely aligned with those observed among the donors. Furthermore, the mice exhibited similar growth performance trends and pathological changes, suggesting that the gut microbiota plays a pivotal role in shaping the phenotype of IUGR piglets and mediating the effects of ICA intervention. Changes in the *Catenibacterium* genus precisely mirrored the donor phenotype. Recent studies have indicated that an increased abundance of *Catenibacterium* might impact pregnancy outcomes and is strongly associated with the occurrence of IUGR [35]. This association has been consistently observed across multiple human cohort studies from diverse regions [36, 37], as well as in pig research [16]. However, the specific functions of *Catenibacterium* remain largely unexplored.

To clarify the biological function of *Catenibacterium* and explore the mechanism of its response to ICA, we selected *C. mitsuokai*, the only known species of the genus *Catenibacterium* [38], for functional experiments. ICA showed a distinct inhibition to the *C. mitsuokai* strain. Following ICA treatment, genes related to ribosomal function and DNA replication were abnormally upregulated in *C. mitsuokai* strain, and those involved in starch and sucrose metabolism were downregulated. These findings suggest that ICA treatment disrupts transcription, translation, and glucose utilization and storage in *C. mitsuokai*, ultimately leading to an imbalance in energy supply. Previous studies have shown that antibacterial antibiotics typically exert their effects by rapidly enhancing bacterial transcription and translation, and simultaneously reducing ATP synthesis. This combined action weakens to sustain basic metabolic activities, ultimately leading to bacterial death [39], a mechanism that has also been demonstrated in *E. coli* and *Streptococcus pneumonia* [40, 41]. Furthermore, the underdeveloped intestine in IUGR piglets results in a reduced starch digestion rate in the ileum, which not only affects their nutritional status but also provides abundant fermentable substrates for the colonic microbiota [42], thereby significantly altering the microbial composition [43]. The suppression of starch and sucrose metabolism in *C. mitsuokai*-like bacteria by ICA aligns with the functional predictions of colonic microbiota in ICA-treated IUGR piglets, and oral administration of *C. mitsuokai* in mice confirms its colonization in the colon rather than the ileum. These observations suggest that the overgrowth of *Catenibacterium* species may deplete the undigested starch in the ileum of IUGR piglets, inhibiting the growth of other beneficial bacteria and ultimately causing an imbalance in the gut microbiota. In line with this, oral administration of *C. mitsuokai* in mice significantly altered the composition of the colonic microbiota, which mirrored the microbial shifts observed in IUGR piglets and contributed to gut barrier dysfunction, accompanied by increased LPS production and translocation. As a Gram-positive bacterium, *Catenibacterium* is unlikely to produce LPS. Instead, our findings suggest that *Catenibacterium* acts as a disruptor of the gut ecosystem, fostering a dysbiotic microbial community that contributes to elevated LPS production.

It has been reported that the increased level of LPS in the serum inhibit Pparα activity in the liver [44]. Our study supports this finding, as oral administration of *C. mitsuokai* in mice resulted in the enhanced expression of Tlr4 and suppressed expression of Pparα in the liver. And similar changes in liver lipid metabolism genes and liver dysfunction in IUGR piglets were also evident in the mice, including compensatory upregulation of *Cpt1a* and *Acox1*, as well as increased *Cd36* expression. Previous studies have shown that Cd36 expression is negatively regulated by Pparα activity, and its elevated levels promote increased uptake of FFA by the liver, contributing to greater lipid accumulation [45]. Both the mice treated with *C. mitsuokai* and the IUGR piglets exhibited elevated FFA levels in the liver, further supporting the role of increased Cd36 expression in lipid metabolism. These liver impairments may influence the synthesis of IGF-1, ultimately resulting in stunted weight gain.

Although microbial metabolites such as short-chain fatty acids and bile acids are known to influence PPAR signaling pathways [46], our study specifically support LPS as a central mediator within the gut–liver axis of IUGR piglets. IUGR piglets consistently exhibit disrupted intestinal architecture and increased epithelial permeability, facilitating LPS translocation. In line with this, our previous study demonstrated elevated hepatic TLR4 expression, a receptor activated by LPS, was significantly upregulated in male IUGR piglets as early as postnatal Day 7 [2]. Moreover, it is well established that elevated circulating LPS suppresses hepatic PPARα expression [44], providing a mechanistic link between gut barrier dysfunction and hepatic lipid dysregulation. Given that ICA functions as a natural agonist of PPARα, we hypothesized that its beneficial effects may involve both direct activation and indirect modulation through reduced LPS burden. Therefore, our data highlight the importance of LPS translocation, rather than other microbial metabolites, as a dominant contributor to PPARα suppression in the IUGR context.

The current research has pinpointed *Catenibacterium* as a significant microbial target by monitoring ICA-induced alterations in gut microbiota and corroborating the results through FMT. Furthermore, experiments performed both *in vivo* and *in vitro* with *C. mitsuokai* have reinforced the role of *Catenibacterium* in facilitating ICA-induced enhancements in hepatic lipid metabolism among IUGR piglets. Considering the intricate nature of the gut microbiome, subsequent investigations should explore co-culture models (for instance, *C. mitsuokai* alongside *Limosilactobacillus*) or synthetic consortia, combined with metabolomics, to clarify the metabolic interactions between microbes and hosts. Moreover, it is essential to conduct long-term follow-ups and broaden trials to evaluate the lasting impacts of early ICA intervention on growth performance, microbiota, and hepatic lipid metabolism in male IUGR piglets.

## Conclusion

**Figure 8:**
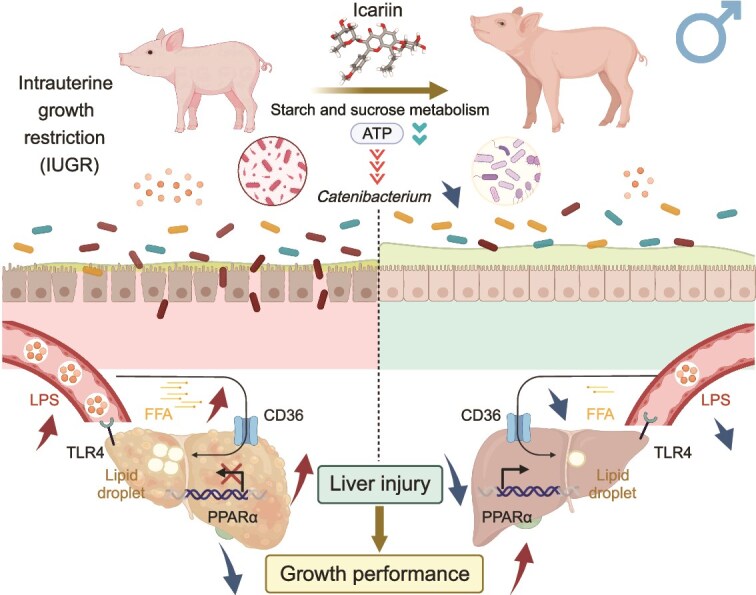
ICA suppresses the proliferation of *Catenibacterium* by inhibiting ATP synthesis. This alteration in microbial composition improves intestinal morphology and barrier integrity, and reduces LPS translocation. Consequently, the hepatic PPARα/CD36 pathway is activated, which helps restrict excessive free fatty acid absorption and triglyceride accumulation, thereby promoting liver health and improving growth performance in male IUGR piglets.

ICA suppresses the proliferation of *Catenibacterium* species, thereby reshaping the colonic microbiota and restoring intestinal barrier integrity, which in turn reduces the production and translocation of LPS. Through the synergistic actions of the gut–liver axis, ICA further activates the PPARα/CD36 axis, effectively reducing hepatic lipid accumulation and, ultimately, improves liver function and growth performance in male IUGR piglets (Fig. 8).

## Supplementary Material

Supplementary_information_wraf141

Table_S1_wraf141

Table_S2_wraf141

## Data Availability

The sequencing data generated in this study have been deposited in the NCBI BioProject database under accession numbers PRJNA1233914 (16S rRNA gene sequencing) and PRJNA1233895 (prokaryotic transcriptome sequencing).
