# Supplementary material for: Targeting Catenibacterium mitsuokai with icariin modulates gut microbiota and improves hepatic lipid metabolism in intrauterine growth restriction
Source: ISME J. 2025 Jul 3;19(1):wraf141. doi: 10.1093/ismejo/wraf141 (PMC12315679; doi:10.1093/ismejo/wraf141)
Supplement: Supplementary_information_wraf141 [file supplementary_information_wraf141.pdf]

Supplementary Materials for

Targeting *Catenibacterium mitsuokai* with icariin modulates gut microbiota and improves hepatic lipid metabolism in intrauterine growth restriction

Yusen Wei[1,#], Jiangdi Mao[1,#], Wenjie Tang[1], Yanfei Ma[1], Jiachen Li[1], Songtao Su[1], Zhixiang Ni[1], Jinhong Wu[2], Daren Liu[2,*], Haifeng Wang[1,*]

[1] College of Animal Science, Zhejiang University, The Key Laboratory of Molecular Animal Nutrition, Ministry of Education, Hangzhou, 310000, China.

[2] The Second Affiliated Hospital of Zhejiang University, Hangzhou, 310009, China.

[#] These authors contributed equally and share first authorship.

*Corresponding author:

H.F Wang and

D.R. Liu

This PDF file includes:

Figs. S1 to S5

Tables S1 to S2

Supplementary figures

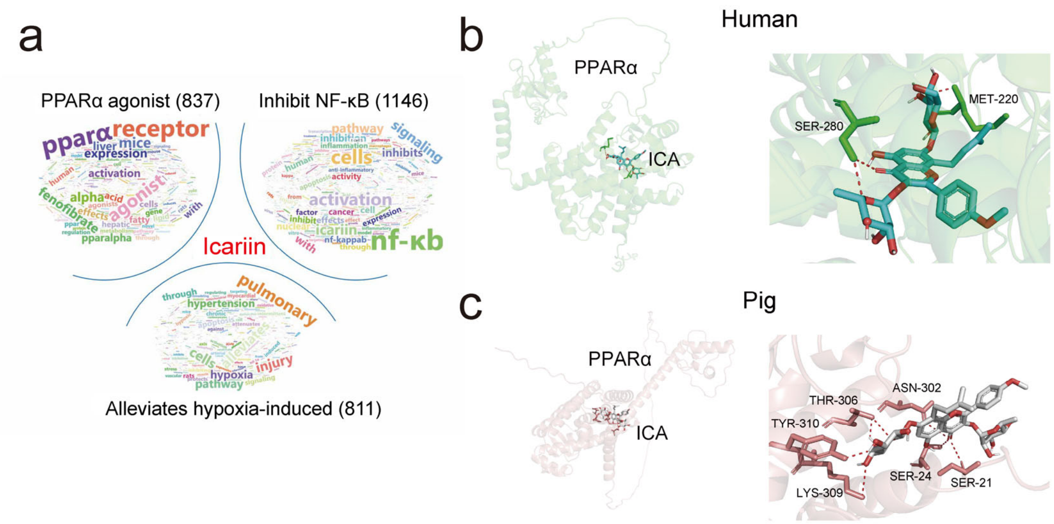

Fig. S1 Literature screening and molecular docking model of ICA with PPARα. (A) Intersection of three major keyword word clouds. (B) ICA with human PPARα. (C) ICA with porcine PPARα. ICA, icariin.

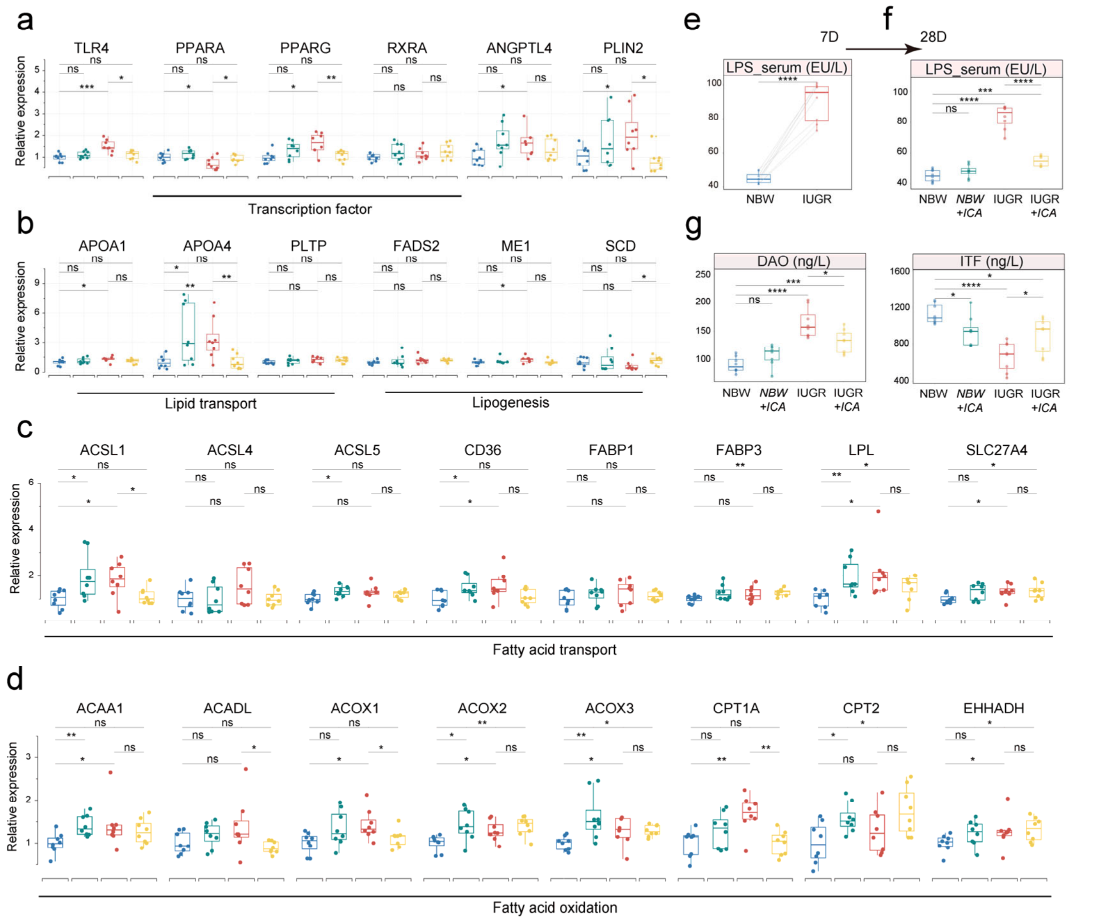

Fig. S2 Changes of ICA intervention on hepatic PPAR signaling pathway and the intestinal permeability of male IUGR piglets. (a) Alterations in mRNA expression of TLR4, transcription factor related genes and lipid droplet marker genes. (b) Alterations in mRNA expression of lipid transport and lipogenesis related genes. (c) Alterations in mRNA expression of fatty acid transport related genes. (d) Alterations in mRNA expression of fatty acid oxidation related genes. (e) The level of LPS in serum at day 7. (f) The level of LPS in serum at day 28. (g) The levels of DAO and ITF in serum. DAO, diamine oxidase; ITF, intestinal trefoil factor; LPS, lipopolysaccharide; ICA, icariin; IUGR, intrauterine growth restriction. The Mann-Whitney U test (a-d, f) and paired Student's t-test (e) (*****P* < 0.0001; ****P* < 0.001; ***P* < 0.01; **P* < 0.05; ns *P* > 0.05).

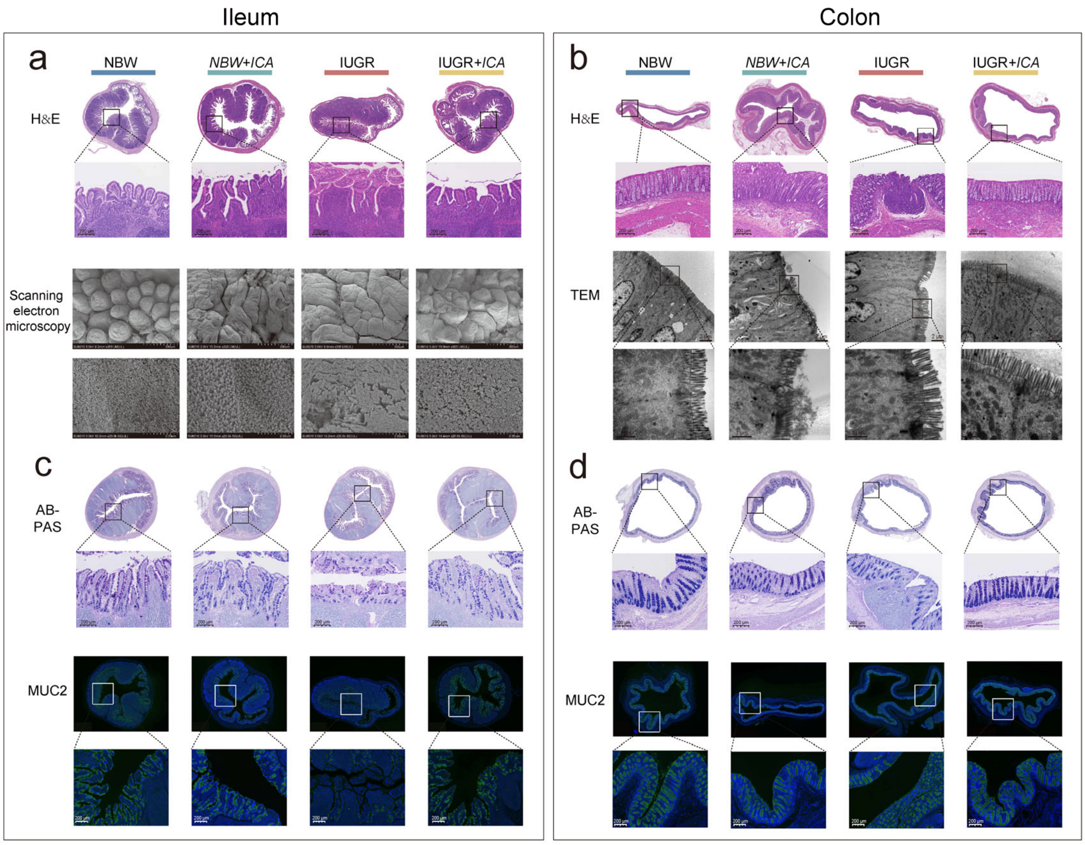

Fig. S3 Changes of ICA intervention on intestinal morphology and function of male IUGR piglets. (a) H&E staining and scanning electron microscopy images of the ileum. (b) H&E staining and TEM images of the colon. (c) AB-PAS staining and IF images of MUC2 of the ileum. (d) AB-PAS staining and IF images of MUC2 of the colon. TEM, transmission electron microscopy; IF, immunofluorescence staining; ICA, icariin; IUGR, intrauterine growth restriction.

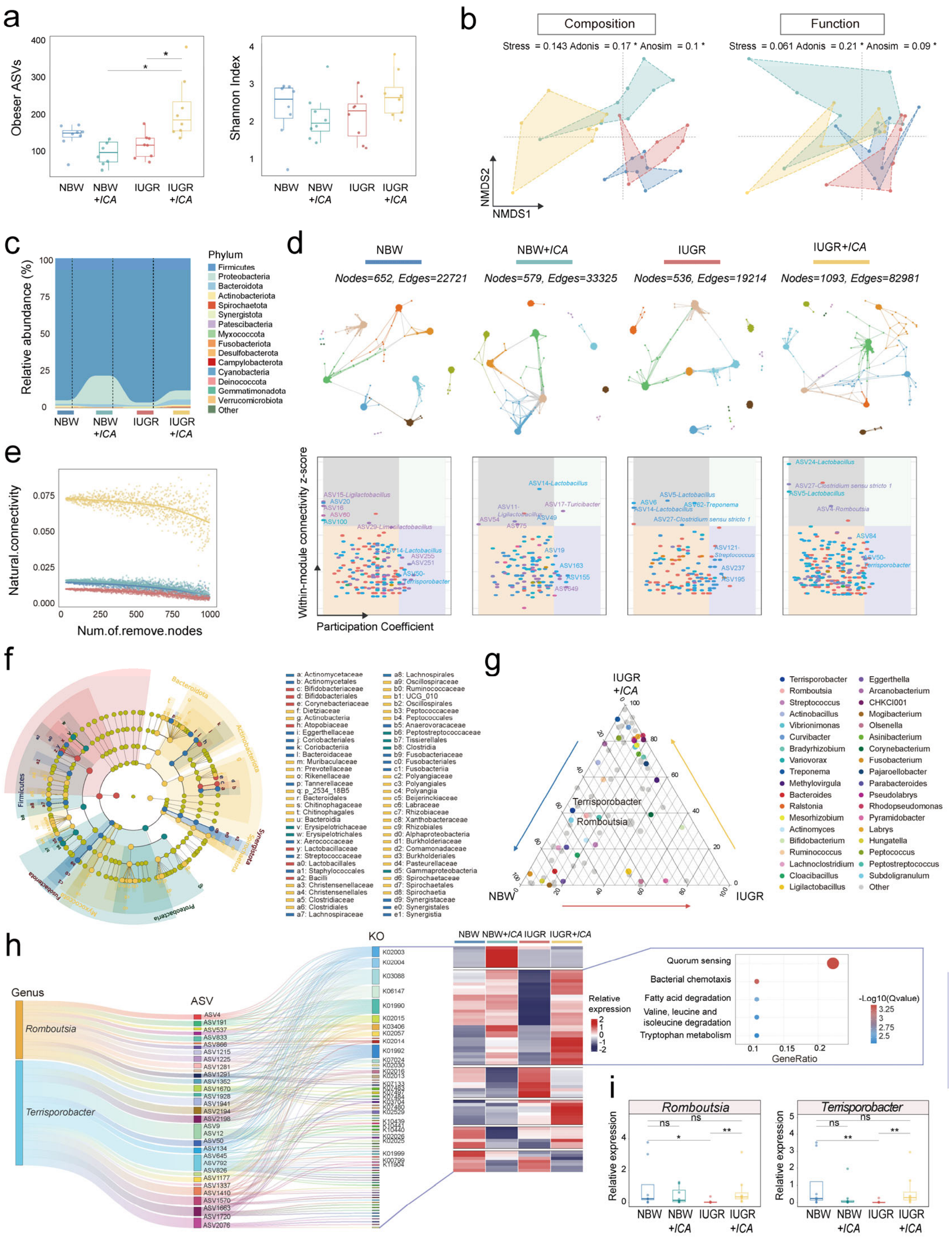

Fig. S4 Changes of ICA intervention on the ileal microbiota of male IUGR piglets. (a) Changes in α-diversity. (b) Changes of β-diversity in microbial composition and function. (c) Changes in microbial composition at the phylum level. (d) Changes in the microbial network. (e) Stability of microbial networks. (f) LEfSe analysis of different microbiota. (g) Ternary analysis of different genera. (h) Functional prediction and enrichment analysis of crucial genera. (i) Changes in relative abundance of crucial genera using RT-qPCR. ICA, icariin; Statistical significance was determined using the Mann-Whitney U test (a, i) (***P* < 0.01; **P* < 0.05; ns *P* > 0.05).

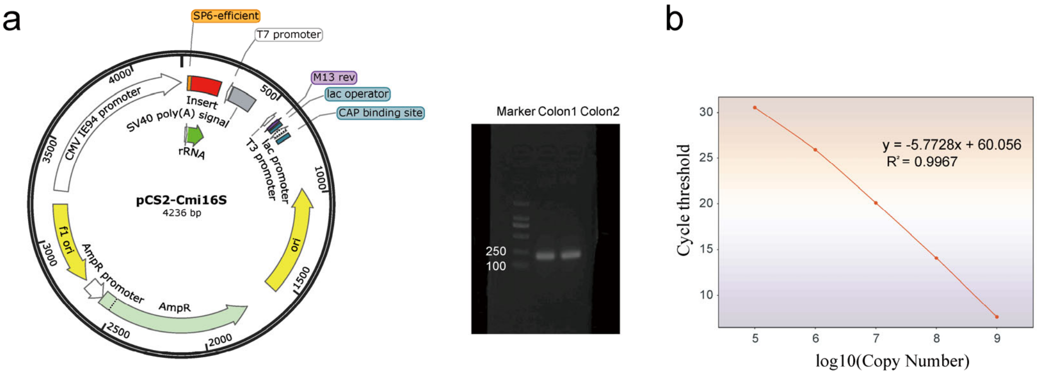

Fig. S5 Construction of the standard plasmid for *C. mitsuokai*. (a) Diagram of the plasmid structure and verification of amplification products. (b) Standard curve.

Supplementary tables

Table S1. Detailed information of primer sequence, antibodies and ELISA kits.

Table S2. Quality control and expression matrix of prokaryotic transcriptome.
